# Migratory Wild Birds as Potential Long-Distance Transmitters of *Toxoplasma gondii* Infection

**DOI:** 10.3390/pathogens12030478

**Published:** 2023-03-18

**Authors:** Filippo Maria Dini, Giulia Graziosi, Caterina Lupini, Elena Catelli, Roberta Galuppi

**Affiliations:** Department of Veterinary Medical Sciences, University of Bologna, Ozzano dell’Emilia, 40064 Bologna, Italyroberta.galuppi@unibo.it (R.G.)

**Keywords:** *Toxoplasma gondii*, wild birds, One Health, PCR

## Abstract

*Toxoplasma gondii* is a worldwide distributed zoonotic protozoan capable of infecting a wide range of mammals (including humans) and birds as intermediate hosts. Migratory wild birds, through interconnecting countries along their flyways, can play a role in the spatial spread of *T. gondii* and could contribute to its sylvatic cycle. Additionally, hunted wild birds used for meat consumption could represent a further source of human infection. To determine the presence of *T. gondii* in wild birds, a total of 50 individuals belonging to the Anseriformes and Charadriiformes orders were sampled during the 2021–2022 hunting season in Northern Italy. Cardiac muscle samples of three Northern shovelers (*Anas clypeata)*, two wild mallards (*A. platyrhynchos*), one Eurasian teal (*A. crecca*), and one Northern lapwing (*Vanellus vanellus*) were positive for the molecular detection of *T. gondii* based on a targeted amplification of the B1 gene. A 14% (7/50) overall positivity was observed in the sampled population. Results from this study suggest a moderate exposure of wild aquatic birds to *T. gondii*, highlighting the importance of a further characterization of *T. gondii* in its wildlife hosts.

## 1. Introduction

*Toxoplasma gondii* is a widespread zoonotic apicomplexan protozoan potentially able to infect all the warm-blooded animal species [1]. The life cycle of this parasite is complex, including definitive and intermediate hosts and several transmission pathways. Millions of unsporulated oocysts are shed in feces by felids, the definitive hosts of *T. gondii*. The parasite can therefore be found in various environmental matrices where, upon sporulation, oocysts become infectious and can remain viable up to several years [2]. This environmentally resistant stage is critical to the success of the parasite’s life cycle, and it shapes the extensive range of its intermediate hosts. The oocysts can be further dispersed by wind, earthworms, arthropods, and water [3]; any warm-blooded animal can therefore become infected by the ingestion of oocysts-contaminated soil, water, or plant tissue [2]. For mammals, a vertical route of transmission is also expected [2]. When infecting an intermediate host, *T. gondii* forms life-long persistent cysts located prevalently in neural and muscular tissues, such as brain, retina, and skeletal and cardiac muscles [4]. Through predation between intermediate hosts, tissue cysts act as a reservoir of infection even in the absence of felids [5,6]. Nevertheless, sexual replication of *T. gondii* and the fecal excretion of its oocysts can only happen in the definitive hosts [2]. Environmental factors such as temperature and humidity can affect the life cycle of *T. gondii* by influencing the survival time of unsporulated oocysts; furthermore, seasonal fluctuation in precipitation rates influence the dispersion of sporulated oocysts [7].

Infections caused by *T. gondii* in wildlife and humans can determine heterogeneous clinical observations. Toxoplasmosis can indeed be fatal or chronic, with disease severity affected by host-dependent or parasite-dependent variables (e.g., individual immune response, species-specific susceptibility, strain virulence, and infective dose) [2]. In avian populations, toxoplasmosis can be particularly concerning for endangered species [8,9,10].

Considering the terrestrial definitive host of *T. gondii*, oocysts are exclusively deposited on land and can therefore disperse in freshwater following heavy rainfalls, owing to the hydrophilic nature of their surface [3,11]; this sheds interest on surveys concerning the occurrence of *T. gondii* infections in wild aquatic species. In aquatic environments, intermediate avian hosts belonging to different feeding groups (herbivores, omnivores, carnivores, and insectivores) appear to be subjected to a similar infection probability [12]. In birds, several aquatic species are known hosts of relevant animal or zoonotic pathogens [13,14,15,16,17], including *T. gondii* [18,19]. Considering the ecology of waterfowl and the specialization as filter-feeders of some species, *T. gondii* infection in these animals suggests the presence of an oocysts-contaminated aquatic habitat. Furthermore, being huntable aquatic birds and a human food source, the consumption of raw or undercooked meat, especially derived from niche products, could represent an underappreciated source of *Toxoplasma gondii* infection [19,20]. Given the scarce available epidemiological data, the present study aimed to assess the occurrence of *T. gondii* in wild aquatic birds hunted in the wetlands of Northern Italy, where wintering migratory individuals from different breeding grounds congregate seasonally.

## 2. Materials and Methods

### 2.1. Population of Interest

**Figure 1:**
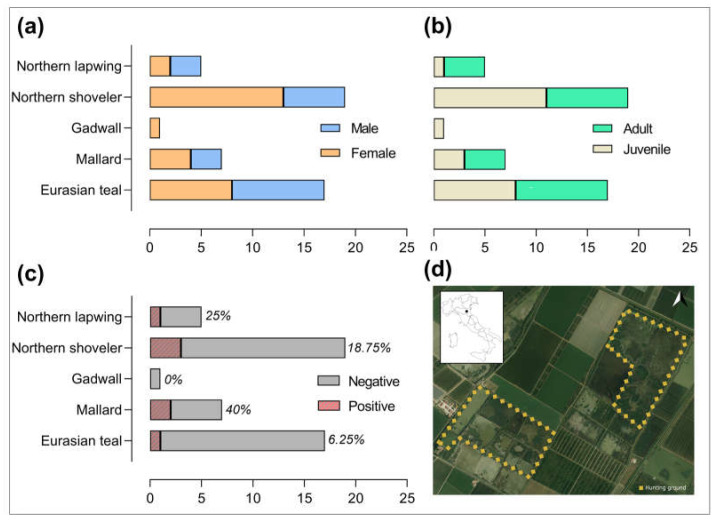
Population of interest, results, and study area. The number of sampled birds according to species and sex (**a**) and age class (**b**); the results of *T. gondii* molecular detection (**c**); and the hunting grounds (**d**), located in the Bologna province, Northern Italy. Map realized with QGIS software version 3.26 [22].

A total of 50 hunted wild aquatic birds were included in this study, selected among 124 individuals sampled within the application of the National Avian Influenza (AI) Surveillance Plan 2021 (https://www.izsvenezie.it/documenti/temi/influenza-aviaria/piani-sorveglianza/piano-nazionale-influenza-aviaria-2021.pdf, accessed on 22 October 2021) and the Commission Delegated Regulation (EU) 2020/689. As an inclusion criterion, only individuals whose entire carcass was preserved were included in the molecular survey hereby presented. Sampling activities were conducted from October 2021 to January 2022 in two hunting grounds (Figure 1d) of the province of Bologna, Emilia-Romagna region, Northern Italy, in an area where wintering migratory birds congregate and intermingle with resident populations. Licensed hunters made available their hunting bags for AI surveillance purpose, and the samplings were performed on the behalf of the Local Health Authority A.U.S.L. of Imola (BO). Birds were hunted according to the National Hunting law 157/1992, without the necessity of any additional permits. Overall, 19 Northern shovelers (*Spatula clypeata*), 18 Eurasian teals (*Anas crecca*), 7 mallards (*A. plathyrhynchos*), 5 Northern lapwings (*Vanellus vanellus*), and 1 gadwall (*Mareca strepera*) were sampled. The sex and age class (juvenile of the year or adult) of each individual were recorded by trained ornithologists, as reported in Figure 1a and 1b, respectively. 

### 2.2. Molecular Detection of Toxoplasma gondii

Heart tissue samples were collected and stored at −20 °C until processing. Genomic DNA was purified from 25 mg of tissue using the Pure Link^®^ Genomic DNA Mini kit (Invitrogen by Thermo Fisher), according to the manufacturer’s protocol. Nested PCR targeting the glycerol-3-phosphate dehydrogenase gene (B1) of *T. gondii* was performed with minor modifications of the protocol described by Jones et al. [21]. Briefly, the first round of amplification included a denaturation step at 96 °C for 2 min, followed by 40 cycles at 93 °C for 10 s, 57 °C for 10 s, and 72 °C for 30 s. The second round was as follows: denaturation step at 95 °C for 2 min, followed by 40 cycles at 93 °C for 10 s, 62.5 °C for 10 s, and 72 °C for 30 s. Amplifications were performed in a T-personal thermal cycler (Biometra, Goettingen, Germany). Amplicons from the first and second PCR rounds have an expected length of 193 bp and 96 bp, respectively. Water was used as a negative control, and *T. gondii* positive DNA was added as a positive control. PCR products were electrophoresed on 2% agarose gel stained with SYBR Safe DNA Gel Stain (Thermo Fisher Scientific, Carlsbad, CA, USA) in 0.5× TBE. For sequencing, the amplicons were excised and purified by Nucleo-Spin Gel and PCR Clean-up (Macherey-Nagel, Düren, Germany) and sequenced with an ABI 3730 DNA analyzer (StarSEQ, Mainz, Germany). Consensus sequences were compared with published sequences by a BLAST search (https://blast.ncbi.nlm.nih.gov/Blast.cgi, accessed on 21 December 2022).

### 2.3. Statistical Analyses

Data analyses were performed using GraphPad Prism (version 8.0) software (GraphPad Software Inc., San Diego, CA, USA). Fisher’s exact test (two sided) was used to determine the association between the molecular results and the species, age class, and sex of the birds tested. A *p* value below 0.05 (*p* < 0.05) was considered significant.

## 3. Results

**Figure 2:**
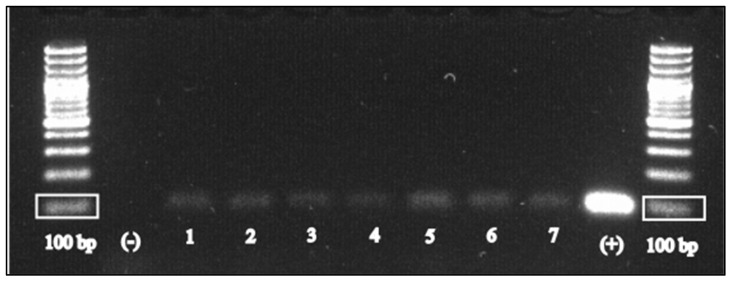
Agarose gel showing the 96 bp amplicons obtained from the individuals tested positive for *T. gondii*. The following order of samples is shown: negative control, 3 Northern shovelers (slots numbered 13), 1 Northern lapwing (slot 4), 1 Eurasian teal (slot 5), 2 mallards (slots 6 and 7), and *T. gondii* positive control.

The amplification of the B1 gene was successful in 7 out of the 50 heart specimens tested (14%) (Figure 2). BLAST searches on the obtained sequences gave a 100% identity with *T. gondii*. Sequence data were submitted to the NCBI GenBank database under the following accession numbers: OQ646717-23. As shown in Figure 1c, 3 Northern shovelers (1 juvenile female and 2 adult males), 1 Eurasian teal (adult male), 2 mallards (1 juvenile female and 1 adult male), and 1 Northern lapwing (juvenile male) tested positive. There was no association was observed between the molecular detection of *T. gondii* and species (*p* > 0.1), sex (*p* = 0.43), or age class (*p =* 0.09) of the birds tested.

## 4. Discussion

Considering *T. gondii* oocysts as widely distributed in the environment, especially in aquatic habitats [23,24], this study aimed to assess the molecular occurrence of *T. gondii* in hunted wild aquatic birds. The population tested included species with different habits and migratory strategies. As a result, parasitic DNA was found in 14% (7/50) of the heart samples tested, showing a moderate exposure of waterbirds to the infection.

*Toxoplasma gondii* molecular detection in individuals belonging to the Anseriformes order, namely, Northern shovelers, mallards, and Eurasian teals, confirmed previously published records for other geographic areas surveyed [16,25,26,27,28]. From an ecological point of view, the above-mentioned dabbling ducks are long-distance migrants along the Black Sea–Mediterranean flyway, which also encompasses the Italian wetlands. Mean estimates from ring recoveries data demonstrated that Eurasian teals and mallards can displace up to 326.5 km and 289.63 km per day during migration, respectively [29]. Epidemiological surveys carried out in wild and domestic animals sampled in North-Eastern Europe and Russia, where dabbling ducks’ breeding grounds are located, reported serological or molecular detection of *T. gondii* in different intermediate hosts, suggesting a possible local infection [18,30,31]. Wild ducks could get infected since hatching and, through seasonal migratory movements between wintering grounds located in Italian wetlands and their breeding territories, could therefore play a role as long-distance transmitters in the *T. gondii* epidemiology. 

For Northern lapwings, a species belonging to the Charadriiformes order commonly found in open lands and mudflats, *T. gondii* infection has already been reported by Nardoni et al. [26] in central Italy. Although considered migratory in other areas, lapwings are usually residents in southwestern Europe [32]; it is therefore likely that the adult individual hereby tested positive was locally exposed to the parasite, like the juvenile ducks (2 out of the 6 ducks tested). Wetlands in Northern Italy are represented by natural areas interspersed with anthropic environments used as water storage for cropland irrigation, hunting grounds, or wastewater plants. The environmental contamination of freshwater with *T. gondii* oocysts could be associated with inefficient sewage treatment, water discharge, and water runoff, as already demonstrated for marine environments [33]. Considering the earliest arrival of migratory ducks in Italian wintering grounds in August [34] and the *T. gondii* detection in the studied population since mid-October, local exposure to the parasite may also be likely for the adult ducks. In fact, an experimental study in chickens reported the detection of tissue cysts in brain, heart, liver, spleen, or lungs, starting from 7–15 days post-infection [35]. 

Molecular studies aimed at the detection of *T. gondii* in wild birds have been carried out testing different matrices [36]; in this survey, heart samples only were collected. These are widely used for *T. gondii* PCR detection, even in wild aquatic birds [18,25,26], and can be associated with different techniques of DNA extraction developed to improve parasite detection and quantification [37,38]. Although the DNA extraction technique adopted in this study involved the use of a small amount of tissue, potentially resulting in less sensitivity than others such as magnetic-capture DNA extraction, higher overall molecular positivity (14%) has been hereby observed in comparison with previous records in wild aquatic birds [16,25,26,39,40,41,42,43]. For Italy, Mancianti et al. [25] and Nardoni et al. [26] performed serological investigations in waterbirds, and seropositive-only individuals were tested by PCR. As a result, 8.7% [25] and 8.1% [26] of the hunted waterfowls were seropositive and, among these, *T. gondii* was molecularly detected in 3 out of 9 and 8 out of 12 individuals, respectively. However, as reported by Opsteegh et al. [44] for *T. gondii* infection in cattle, also seronegative animals could harbor tissue cysts. The loss or absence of a detectable antibody titer in infected individuals has been observed in several bird species [45,46], while Bachand et al. [39] found discrepancies between serological and molecular results in both directions (e.g., seronegative animals with *T. gondii* positive tissues, and seropositive animals with tissues negative for parasite DNA). The molecular screening of all the individuals studied, as performed in this survey, could therefore reflect the actual percentage of birds that harbor tissue cysts. Furthermore, although no correlation with sex and age was hereby observed, these variables deserve attention in further investigations with a larger sample size to obtain a better understanding of *T. gondii* epidemiology in wild aquatic birds. 

From a One Health point of view, the detection of *T. gondii* in heart samples may indicate the presence of tissue cysts in edible muscles, such as the pectoral ones, whose consumption could determine a meat-borne transmission of the parasite. In addition, it has been shown that handling wild or domestic animal carcasses without appropriate hygiene practices could lead to infection due to the accidental contamination of hand or other equipment from bradyzoites released from tissue cysts during cutting practices [47,48,49,50]. 

To conclude, results from this study highlight moderate exposure to *T. gondii* in wild aquatic birds from Northern Italy, suggesting their importance as biological indicators for *T. gondii* contamination of aquatic habitats and their potential contribution to the sylvatic cycle of the parasite. Furthermore, migratory species could act as *T. gondii* long-distance transmitters, considering their ecology and habits. An additional genetic characterization of the positive samples is needed to establish the role of migratory birds in linking countries where different genotypes circulate.

## Data Availability

GenBank accession numbers: OQ646717-23.
